# Oncology nursing on the move: a contemporary issue on Chinese oncology nursing in cancer care

**DOI:** 10.3389/fpubh.2023.1061572

**Published:** 2023-04-27

**Authors:** Yingyang Li, Wenjing Yu, Lamei Li, Qianqian Yao, Kexin Jiang, Tao Zhu, Enshe Jiang

**Affiliations:** ^1^Institute of Nursing and Health, Henan University, Kaifeng, China; ^2^Department of Orthopedics, Henan Provincial People’s Hospital, Zhengzhou, China; ^3^Department of Neonatal Intensive Care, Zhengzhou Central Hospital, Zhengzhou, China; ^4^Department of Geriatrics, Kaifeng Traditional Chinese Medicine Hospital, Kaifeng, China; ^5^Henan International Joint Laboratory for Nuclear Protein Regulation, Henan University, Kaifeng, China; ^6^Department of Scientific Research, Scope Research Institute of Electrophysiology, Kaifeng, China

**Keywords:** cancer, health system, Chinese nurses, oncology nursing, nursing education

## Abstract

Cancers have become the primary cause of death among Chinese residents, seriously affecting their health and life. Oncology nursing is a specialized nursing practice focusing on cancer education, prevention, screening, early detection, and palliative and hospice care. China has made tremendous progress in developing oncology nursing. However, to ensure more individuals can get cancer care, the country’s healthcare system still confronts several problems in oncology nursing that need to be addressed to ensure that more individuals can receive cancer care. This article reviews the current development of oncology nursing in China, especially in pain symptom control, palliative care, end-of-life care, education and training. The challenges faced in oncology nursing in China and the suggestions for developing oncology nursing in China are also discussed and proposed in this review. The growth of research on oncology nursing by Chinese nursing scholars and concerned policymakers is anticipated to ultimately improve oncology nursing and the quality of life of patients with cancer in China.

## 1. Introduction

Cancer has often been considered the most devastating disease, and the number of patients requiring cancer care is escalating rapidly. A global oncology nursing workforce is essential to meet the cancer care need. Retaining experienced oncology nurses is crucial for cancer control in cancer care units, especially in resource-constrained countries with limited oncology nursing staff (1). In addition, oncology nursing is a fundamental need of healthcare units as cancer prevalence increases. 18.1 million people are diagnosed with cancer each year, of which 9.6 million cancer patients die across the globe, and this incidence rate will reach 29.5 million by 2040 (2, 3). As the initial point of contact for patients, the oncology nurse performs a range of roles throughout a patient’s cancer treatment. Although cancer patients may seek help from other caregivers to cope with their diseases, nurses are frequently involved in the patient’s care throughout their illness. In clinical practice, a patient’s first interaction with the health system is with a community health worker at a dispensary. Therefore, with sufficient medical education and better training, the nursing department is considered a central pillar in the health system, especially in oncology. In addition, nurses can create awareness of cancer and its risk factors, signs and symptoms (4).

**Figure 1:**
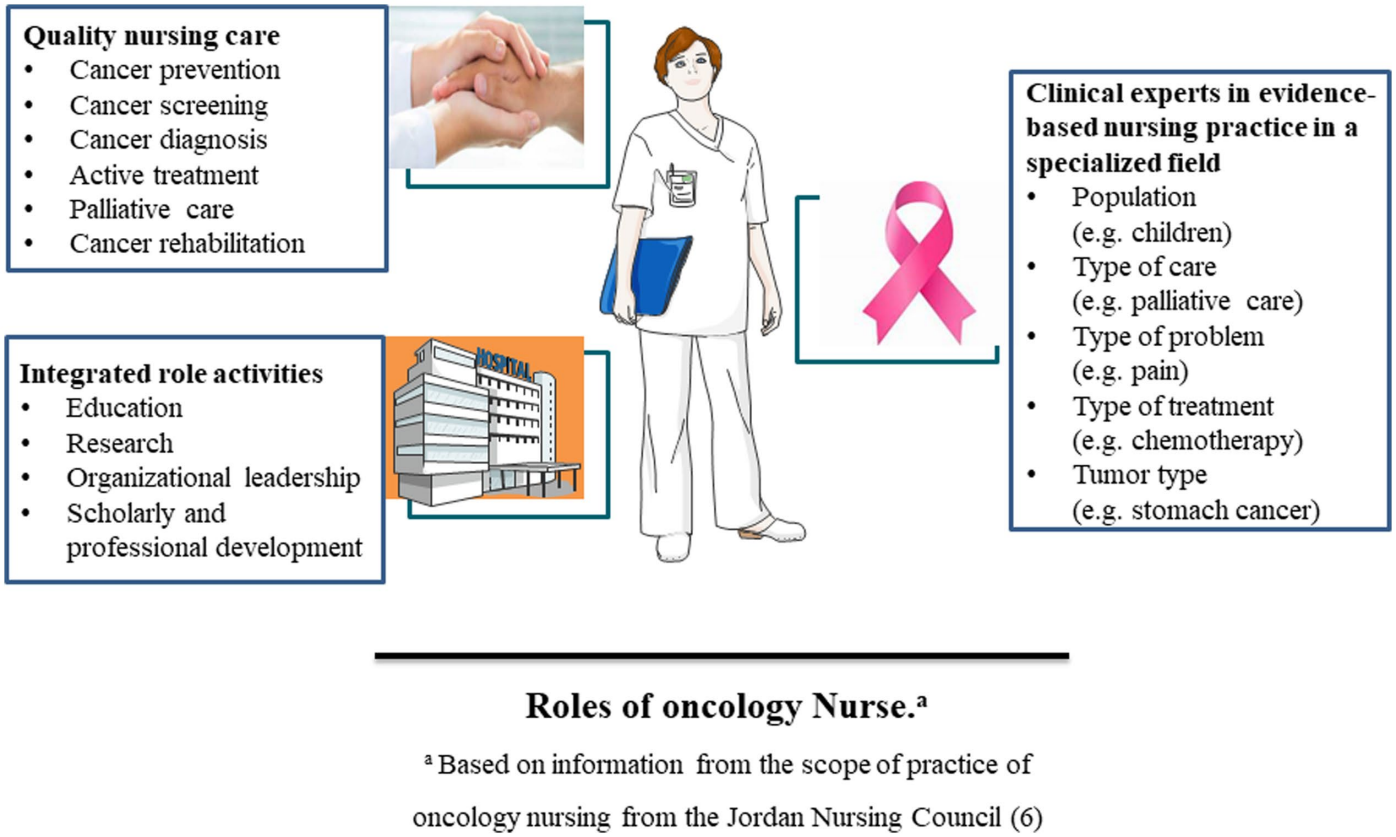
The oncology nurse’s role in quality nursing care, integrated role activities, and clinical experts in evidence-based nursing practice in a specialized field.

Oncology nurses can provide high-quality care for oncology patients, especially in oncology prevention, screening, diagnosis, active treatment, palliative care, and rehabilitation care. They also play a crucial role in collaboration with a multidisciplinary medical team (5). Oncology nurses are clinical experts in evidence-based nursing practice in a specialized field. This specialty may focus on a certain population (such as children), type of care (such as palliative care), type of problem (such as pain), type of treatment (such as chemotherapy), or type of tumor (such as stomach cancer). In addition, oncology nurses play an essential role in the integrated activities of education, research, professional development, organization and leadership of oncology nursing, which can effectively promote the development of oncology nursing practice and healthcare delivery (6). The role of oncology nurses is dynamic and up-to-date. Their role is to maintain the patients with cancer in the maximum possible state of health. The roles of the nurses in the oncology setting are summarized in Figure 1.

A robust oncology nursing workforce is essential for effective service as it demands advanced diagnostic and treatment tools and specialized medical oncologists, particularly pathologists and radiation oncologists. The most constructive intervention leading to better outcomes is to develop oncology training and learning for comprehensive education (7). It holds a great promise to meet the basic educational needs about the disease, the training of the healthcare force, and awareness of the subject in the general population (8).

Oncology nurses perform a pivotal role in the healthcare system via their commitment to strengthening oncology practice to support the quality of services. With these high cancer death rates and the imbalances in the global health workforce, this review summarizes the development of Chinese oncology nursing and the pivotal role of oncology nursing in cancer care. It also discusses the challenges and suggestions for developing oncology nursing in China to emphasize the need to establish oncology nursing in the Chinese medical health system and its impact on cancer care. Considering the need for and importance of the topic, we anticipate that the review will promote the urgency of establishing specialized oncology nursing in cancer clinics.

## 2. A global outlook of oncology nursing

The global oncology nursing workforce is essential to achieve Sustainable Development Goals 3.4 (reduce non-communicable disease morbidity by a third till 2030) and 3.8 (universal health coverage). However, the knowledge, talents, and competencies necessary for these professions entail a higher level of training and education than what can be observed today (9). According to the World’s Nursing 2020 report, there are 19.3 million people in the global professional nurse workforce. However, in 2018, there was an estimated global shortage of 5.9 million nurses, 89% in low- and middle-income countries (LMICs) (10).

Due to the poor economic status, LMICs lacked the amount of sufficiently trained oncology health workers: only 1.3 physicians with 2.5 nurses were available per 1,000 people, as compared to the high-income countries, where 3.1 physicians and 10.9 nurses are allocated for the care of 1,000 cancer patients (11, 12). The high number of patients and the resulting clinical burden for oncology clinicians paired with inadequately trained oncology medical staff pose substantial hurdles to cancer care and further increase cancer-related deaths in LMICs (13, 14).

Several organizations have developed oncology nursing competencies and curricula to prepare competent oncology nurse specialists. These organizations include the European Oncology Nursing Society, the Oncology Nursing Society, the Association of Pediatric Hematology/Oncology Nurses, the Canadian Association of Nurses in Oncology, and the World Health Organization (WHO) Europe. Although these competencies and curricula are based on cancer nursing practice in high-income countries, they may be used to develop competencies and curricula in LMICs (15).

Despite the initiatives of many global oncology programs and policies to organize, promote, and support local oncology education and training, the perpetual imbalances in the global health workforce are the main issues for improving local capacity to provide high-quality cancer treatment (14). WHO strives to develop ties across institutions to support bidirectional learning and knowledge transmission, promote capacity growth, and adapt e-learning approaches to oncology education. Some challenges to a robust oncology nursing workforce include nursing shortages, recruitment barriers, and the establishment of oncology nursing in cancer care units (16).

## 3. Cancer burden in China and oncology nursing

With the largest population in the world, China has made extensive progress in promoting health since the 1950s. However, the changes in lifestyles, age structure, and living conditions in the Chinese population have led to a shift in disease burden from infectious to non-communicable diseases (NCDs). As a result, cancer has become the leading cause of death in China (17). According to the National Central Cancer Registry of China, approximately 2.338 million cancer deaths occurred in 2015 (18). Over the past 35 years, the incidence and mortality rates of liver cancer, stomach cancer, esophageal cancer, and cervical cancer have stayed high, while those of lung cancer, breast cancer, colorectal cancer, and prostate cancer has been growing very fast in China (19–22). Thus, an effective cancer control system must be established and refined in line with the current socioeconomic status of the country. Cancer prevention and control strategies implemented in the US and the UK are valuable references for China (23).

Although China has revolutionized its health system, it still lacks established oncology settings to deal with the increasing cancer burden in the country. In addition, studies about Chinese oncology nurses are lacking. A multicentre study of oncology nursing job satisfaction in China noted a severe shortage of nurses in general, which required oncology nurses to deliver high levels of primary patient care, thus, precluding their ability to address individual patient needs (24). The Chinese Nursing Association or individual hospitals run oncology nurse training programs in mainland China. However, there are no unified criteria for the accreditation of oncology training organizations or unified administrative departments for the accreditation of oncology specialty nurses (25). A recently published study by Li et al. investigated the knowledge and attitudes of Chinese oncology nurses concerning cancer pain management. However, in their study, the survey was administered during an education course on cancer symptom management; thus, participating nurses’ motivation and knowledge levels may differ from those of nurses working in cancer wards (26). Therefore, further investigation is needed to address this gap and guide administrators in equipping oncology nurses accordingly and improving the quality of care they provide to patients with cancer pain. In the following sections, we briefly describe the status and problems in the development of oncology nursing in China and some suggestions were proposed for it. This review will provide a reference for the development of oncology nursing and improvement in the quality of life of patients with cancer in China.

## 4. The development and practice of oncology nursing in China

### 4.1. The development of oncology nursing in China

As early as the 1930s, there was an oncology department at Peking Union Medical College. Founded in 1931, Shanghai Zhongbi Leding Hospital is the earliest specializing hospital in treating tumors in China. It is the predecessor of Shanghai Tumor Hospital. Tianjin People’s Hospital established its oncology department in 1952, and now it has become Tianjin Tumor Hospital and Institute of Oncology Research. The Chinese Academy of Medical Sciences founded the first tumor hospital in 1958. It was renamed the Institute of Oncology Research and Tumor Hospital In 1961 (27). Since then, tumor hospitals or oncology research institutes have been established in provinces, cities and some areas with high cancer incidence. Oncology departments have also been established in general hospitals. The development of the tumor hospital promoted the development of specialized oncology nursing. In recent years, there have been many public or private tumor rehabilitation hospitals nationwide (28). In order to promote the development of oncology nursing, the surgical care group of the Chinese Nursing Association organized and held the first National Oncology Nursing Conference in 1987. The Chinese Nursing Association formally established the oncology nursing professional committee in 1989. It became a group member of the International Society of Nurses in Cancer Care in 1990, the only professional committee for the Chinese Nursing Association to participate in international organizations. Each province and city organized the training class on oncology nursing progress accordingly. They organized the new technology and academic exchange in oncology nursing, creating a vibrant academic atmosphere for oncology nursing (29). In the early 21st century, Oncology nursing has already developed into specialized oncology nursing.

### 4.2. Clinical status of oncology nursing in China

The current service areas of oncology nursing are expanding and intersecting with many disciplines. The oncology treatment consists of many approaches, including surgery, chemotherapy, radiotherapy, and immunotherapy. In each of these treatments, oncology nurses play a significant role. In addition, symptom control and supportive therapy have become integral to the cancer management plan. The oncology nurses also play a critical role in managing the disease itself and the side effects of treatment (30). The oncology nurses provide a range of supportive care measures to cancer patients, such as pain management, nutritional support, and skin care. From diagnosis to hospitalization, treatment and discharge, oncology nurses deliver personalized, holistic services that cater to patients’ individual needs while fully respecting the patient’s personality and rights (31). The oncology nurses combine basic and specialized nursing practices depending on patients’ individual needs, optimizing treatment effectiveness and minimizing the side effects of chemotherapy and radiotherapy. In clinical practice, oncology nurses offer quality care and assist patients in navigating through the difficulties of life. During the perioperative period, oncology nurses provide psychological support, health education, and rehabilitation guidance to help patients understand their illnesses and positively engage in recovery plans. Upon discharge, oncology nurses provide patients with clear instructions on contacting them, when to schedule follow-up appointments, and what precautions to take about medication, diet, and physical activity. Currently, with the establishment of specialized tumor hospitals in most cities throughout China, in addition to the oncology departments of large tertiary hospitals, the needs of cancer patients are met to a significant extent.

### 4.3. Status quo of community oncology nursing in China

Community care for patients with tumors in China draws on the models applied by European and American countries. However, due to differences in policies, general cognition, quality of medical personnel and economic conditions, there are significant differences in community care between China and European and American countries. Unified policies and norms have not been formed in different regions in China. The Central Committee of the Communist Party of China and The State Council issued “the Opinions on Deepening the reform of the medical and health system” On March 17, 2009. This document proposed “establishing the division of labor and cooperation mechanism between urban hospitals and community health service agency, guiding the diagnosis and treatment of common diseases sinking to a primary hospital or medical institution, and gradually realize the medical system of first diagnosis in community, hierarchical medical treatment and two-way referral” (32). However, the development of the medical system of first diagnosis in the community and two-way referral between large comprehensive hospitals and community health service agencies are still slow after years of exploration and practice. The actual system of first diagnosis in the community has not yet been established in China (33). There are mainly community care models for patients with cancer in China: the medical association model, the continuous service model between hospitals and community institutions, the two-way referral model, the hospital-based tumor follow-up model, and the “hospital-community-family” interactive intervention model (34).

### 4.4. Mode of criteria for training and practice of oncology nurses in China

In the late 1980s, Chinese nursing experts began to put forward the idea of training specialized nurses. In 1989, the Chinese Nursing Association formally established the oncology professional committee. The Chinese nursing experts aimed to accelerate the development of “nursing specialization.” The former Ministry of Health issued “the training program for nurses in the field of specialist nursing” in 2007. The training of nurses in the field of specialist nursing in China began to enter a period of large-scale exploration under the background of formulating the planning outline of nursing development in China (35). oncology nurses have gradually emerged and developed with the frequent application of new drugs and technologies in the field of oncology. As a result, oncology nursing was taken as a critical clinical specialized nursing in China. The national training bases for oncology nursing have been established in many regions in China (36).

The specific requirements for selecting oncology nurses varied from hospital to hospital in China. Most of them are: ① healthy and under 40 years old; ② nursing college degree or above; ③ More than 2 years of clinical practice experience; ④ With the title of a senior nurse or above. Some hospitals or training units have requirements for English level and scientific research ability, such as having an English level of CET-4 or above and more than 1 paper published in core journals. There is no unified training course or content for Chinese oncology nurses. The training for oncology nurses mainly consists of two parts: oncology theory and practice. The theoretical part includes basic knowledge of oncology, such as introduction to oncology nursing, methods and principles of clinical tumor treatment, and knowledge of tumor radiotherapy and chemotherapy. Most of the internships are in the field of oncology (25).

Since the training system for oncology nurses in China has not yet been unified, the qualification certification is also not uniform. Some “oncology nurse certificates” were issued by the Chinese Nursing Association, and some “oncology nurse training certificates” were issued by the provincial health department. Individual hospitals also issued some training certificates. The re-certification system has not yet been implemented in China. Since the specialist nurses in China do not have the formal qualification certification by a unified administrative department, they are not fully recognized by patients, hospitals, society and peers, and their professional value is difficult to reflect (37, 38).

### 4.5. Oncology nurse’s knowledge and attitude in cancer care

#### 4.5.1. Cancer pain

Patients and their families, health care personnel, and the health care system all influence the effect of cancer care (39, 40). Tumor compressing or infiltration near the affected body part leads to an uncomfortable pain in the patient. This pain may arise during diagnostic procedures, treatments, and body immune responses. In care units, cancer pain management is based on the previous treatment and coping strategies, which are directly related to the patient’s history, such as emergency department visits, hospitalization and level of therapy satisfaction (41, 42). Incorrect information concerning pain management and the corresponding attitudes of nurses toward patients who experience pain will impede pain management in patients (43). According to the theory of knowledge attitude/brief practice, knowledge is the basis, belief/attitude is the motivation, and behavior is the objective. Therefore, theoretical knowledge is a critical foundation for oncology nurses practicing (44, 45). While caring for cancer patients with difficult circumstances and multiple systemic problems, oncology nurses need solid knowledge and attitudes about better treatment outcomes.

The knowledge and attitude of oncology nurses significantly impact pain improvement in tumor patients. However, research on the knowledge and attitude of cancer pain treatment among oncology nurses in China shows that oncology nurses frequently lack awareness and attitudes about cancer pain (46, 47). Despite several recommendations and pharmacological and nondrug therapies for cancer pain management, oncology nurses’ education is still not successfully managed in China, and patient satisfaction with cancer pain management is even worse (45). In addition, The studies showed that oncology nurses were unfamiliar with the fundamental concept of pain treatment, the content of the principles for the three ladders of analgesic treatment, the principle for pain evaluation, and adverse responses to the use of opioids (45, 46). Therefore, a baseline assessment of oncology nurses’ cancer pain knowledge and attitudes is critical in China. Furthermore, understanding the factors that affect nurses’ attitudes will help develop strategies and resources they need to use to cope in stressful situations. As a result, more research is needed to close this gap and aid administrators in properly equipping oncology nurses and increasing their quality of care.

#### 4.5.2. Oncology nursing in palliative cancer care

Palliative care improves the quality of life for patients with severe or life-threatening diseases like cancer. All sorts of cancer patients in each stage require palliative care, as do their families (48). According to the WHO, 19.92 million individuals worldwide require palliative care at the end of their lives, with cancer accounting for 34% of these patients (49). These statistics show that many cancer patients die or receive hospital end-of-life care. Therefore, oncology nurses are crucial in caring for these patients during this critical period (50). As a result, oncology nurses must be confident in providing care to dying patients, discussing treatment goals with the patient and family, and having the knowledge and skills to provide end-of-life care.

In recent years, palliative care in oncology nursing in China has developed rapidly and significant progressed. However, there is a severe imbalance in the development of oncology nursing among different regions. Professional palliative care departments have been established in Chinese first-tier cities such as Beijing, Shanghai, Guangzhou, and Shenzhen. However, there were no palliative care departments in some relatively undeveloped regions. Most of the hospitalized patients are terminal cancer patients (51). There are four main ways of palliative care in oncology nursing in China. One is the establishment of palliative care departments in general hospitals, the other is independent palliative care hospitals, the third is the establishment of palliative care wards in cancer hospitals, and the fourth is the family nursing center established in communities (52). Since the development of palliative care in China is still in its infancy, no corresponding law or medical insurance system restricts the development of palliative care in China. Due to the influence of traditional ideas, many patients avoid talking about death, and many patients believe that accepting palliative care is a sign of giving up hope. In addition, the lack of publicity in some media further increases patients’ misunderstanding of palliative care, resulting in a low acceptance rate in China. Patients with cancer are generally not informed of the diagnosis result, which is not conducive to patients’ emotional control and treatment. With the transformation of patients’ thinking and consciousness about cancer, many patients began to accept their condition and plan their follow-up life according to it (51, 52).

Due to the particularity of the work of oncology nurses, they not only master the professional knowledge and skills of oncology nursing but also become familiar with the related knowledge of palliative care to provide a higher quality of life and comfortability for patients with middle and advanced malignant tumors (53). However, Chinese oncology nurses have not fully received standardized training in palliative care education (54). Studies showed that the cognitive level of palliative care for most nurses working in the oncology department was at the middle or lower level (55, 56). Zou (57) found that nurses had little knowledge of palliative care’s social, spiritual and psychological aspects. On the other hand, the research results from Liu et al. (53) showed that nurses in the oncology department had a higher demand for palliative care knowledge, an active learning attitude and a comprehensive grasp of knowledge. These results indicate that oncology nurses can better understand patients in their situations, which is one of the reasons for a better understanding of the connotation of palliative care. Through a series of cognitive interventions in palliative care, the cognitive level of nurses in palliative care will be gradually improved and their attitude will be more positive. These will help patients with cancer spend the terminal stage of the disease smoothly, serenely and comfortably (58).

#### 4.5.3. Oncology nursing attitude toward end-of-life

China is responsible for roughly 22% of all new cancer cases globally (59). According to recent cancer statistics, China will have 4.3 million new cancer cases and 2.8 million cancer deaths yearly. Around 10,000 people in China are diagnosed with cancer daily, equivalent to 7 instances every minute (19). Commensurate oncology nursing must match this rapid increase in cancer incidence and mortality in palliative care, end-of-life care, and pain management. However, even though cancer is the leading cause of death in China, little was known about cancer-related end-of-life care until recently, when it began receiving increasing attention (60, 61).

Oncology nurses’ attitudes regarding end-of-life care are directly connected to the patient’s quality of life. However, there are no clear findings on the factors related to oncology nurses’ actions toward end-of-life care, and a theoretical framework needs to be developed. Death is taboo in traditional Chinese society, making communication between nurses, patients, and families difficult (62). As a result, nurses may experience discomfort and repeated setbacks when caring for end-of-life patients, which can harm their physical and emotional health and reduce the quality of end-of-life care they provide (63). Therefore, nurses must be trained to provide physical and mental healthcare, including family care. In addition, they must learn how to maintain patients’ dignity, foster happier and better communication between family members and cancer patients, share knowledge about life and death, and offer ways to cope with negative emotions during care (64).

Data on oncology nurses’ attitudes to caring for end-of-life patients in China are scarce. In addition, most studies’ instruments are self-designed, indicating a lack of systematic and comprehensive assessment criteria and methods. Despite significant advances in other countries, end-of-life care in China is still in its infancy; just a few quantitative studies have examined Chinese nurses’ attitudes regarding caring for terminal cancer patients (65). This study’s findings may provide a basis for improving end-of-life care behavior among oncology nurses, leading to the development of interventions to improve the quality of life of cancer patients at the end of life in China.

## 5. Challenges facing the development of oncology nursing in China

### 5.1. Limited facilities to offer hospice and palliative care challenges

As the Chinese population ages and the number of patients with life-threatening conditions rises, the number of hospice and palliative care facilities are far from matching the growing need for hospice and palliative care services. Several facilities have provided diverse types of hospice and palliative care services in many provinces over the last few decades. However, hospice and palliative care were not incorporated into the mainstream healthcare system. For now, beds for hospice and palliative care are in considerable shortage, and many patients prefer to die at home. Most hospice and palliative care are still given at secondary and tertiary institutions where high-quality care is provided. So, more hospice and palliative care treatments are needed outside traditional hospice or hospital settings. Government backing for community-based hospice and palliative care services are now the focus of growth in the coming decade, along with other appropriate services, and guarantees that patients may get ongoing treatment in oncology settings.

Medicines are readily available in cancer hospitals and associated oncology settings in big cities to relieve pain or cancer-related pain. However, many patients in rural and isolated regions have little or no access to opioids. To overcome this issue, the government has set a strategy to boost access to palliative care so that the drug should be readily available even in backward or rural areas. The operational mechanism and monitoring laws are being implemented to achieve this goal.

### 5.2. Educational challenges

Although there are currently four levels of oncology nursing education in China, including technical secondary school, junior college, undergraduate, and postgraduate, there is a lack of authoritative and scientific goal guidance for oncology nursing talent cultivation at all levels. The criterion of the final evaluation for the nurses’ admittance to the medical institutions and engaging in oncology nursing is based on passing the qualification examination of the national nurse practicing. This measure does not highlight the differences in cultivating oncology nursing talents at each level (66). The scale of talent training at the graduate level for oncology nursing is small, and the overall quality is not high. Advanced oncology nursing talents with a high theoretical level in teaching, scientific research, clinical practice, and organizational leadership are in short supply nationwide. Although oncology nursing-related courses are currently offered in various nursing schools in China, most are offered as elective courses, which are underappreciated and valued, limiting the development of specialized oncology nursing talents (67). In addition, there are insufficient resources for high-quality oncology nursing teachers in Chinese colleges and universities, and there are few opportunities for oncology nursing teachers to have clinical training. Therefore, they lack training in the new technology and developments in oncology nursing, which has inevitably led to a disconnect between their teaching and clinical practice, further hindering the progress of oncology nursing in China.

Chinese nursing students’ clinical practice has two ways: clinical internship and clinical practice. The practical ability of clinical nursing is the core of nursing talent cultivation. However, the high-quality clinical practical teaching resources for oncology nursing are limited. The conditions for the internship to practice on the bases of oncology nursing and the teachers’ teaching abilities in oncology nursing are uneven. The teachers cultivate the internship in the practical clinical ability of oncology nursing without unified standards, which further brings difficulties to improving the practical clinical ability of oncology nursing students (68). In Chinese hospitals, there is a relative lack of training and continuing education for oncology nurses. In addition, the role of oncology specialist nurses in China is rapidly expanding. Currently, the professional titles of oncology nurses in China often do not reflect their roles and abilities. Furthermore, professional certification institutes are still in shortage (69).

### 5.3. Cultural challenges

Chinese people find it difficult to discuss death, believing that it may disrupt their inner equilibrium, although death is an indisputable fact. Furthermore, there is a misconception in China concerning the function of palliative care and hospice. First, many believe that if they are provided palliative care and hospice, it indicates that their doctors have given up on them and they are just waiting to die. As a result, many terminally sick individuals decline this kind of service. Second, sickness and death are unavoidable and necessary parts of life, yet most people are unwilling to confront them. Third, active therapies, including life-sustaining measures, are administered when a cure is unattainable.

Furthermore, in most cases, family members make decisions rather than patients, and patients are frequently excluded from talks about their treatment. This technique causes patients to be confused and misinformed about their sickness and prognosis. As a result, many patients do not have time to say goodbye and depart the world with regrets, which is not in their or their families’ best interests. Thus, better education for patients and the public is required to make the end-of-life experience as peaceful as possible. On the one hand, improving the quality of hospice and palliative care services is critical to demonstrate more positive results to the public. On the other hand, it is necessary to assist patients in receiving proper treatment at the appropriate time, to lead individuals through the process of facing death, and to provide them with all the information they require to make an informed decision about their care.

## 6. The way forward for the development of oncology nursing in China

### 6.1. Strengthen the symptom management of cancer patients and improve their quality of life

During cancer treatment, radiotherapy and chemotherapy often bring severe side effects to patients. Hence, the focus of nursing is to evaluate and manage the symptoms caused by the disease and the adverse reaction of treatment, such as fatigue, pain, sleep disorders, nausea, vomiting, diarrhea and other physical symptoms, as well as anxiety, depression, uncertainty, hopelessness and other psychological symptoms. Some symptoms often appear simultaneously and show a phenomenon of “symptom cluster,” causing great distress to patients and seriously affecting their quality of life of patients. Given the tumor’s complex development and treatment process, oncology nursing focuses on systematic symptom management, assessment, intervention and evaluation of the malignant tumors and treatment-induced symptoms (70).

Since cancer patients have a long treatment period after diagnosis, quality of life has become the final index to evaluate the effect of cancer treatment, nursing and rehabilitation (71). To help cancer patients return to their pre-disease state as much as possible and strive to improve their quality of life, the continuum of nursing care after cancer treatment cannot be ignored (72). In extended care, oncology nurses are required to guide patients to perform functional exercises after surgery to restore their normal self-care ability, help patients readjust their roles in family and society, and create conditions for their return to community and work. For terminal cancer patients, the primary purpose should be to provide comfort, improve the environment and alleviate the pain. Through hospice care, the oncology nurses have the patients maintain good physical function and high quality of life, safeguard the dignity of the patients with terminally ill, and help the patients calmly and painlessly complete the final journey of life (73). In addition to patients’ physical and psychological monitoring, nurses should extend psychological care to cancer patients and their family’s psychological assessment and support.

### 6.2. Restructuring of oncology nursing training and education in China

As an integral part of the healthcare system and crucial in cancer management, Chinese nursing students need quality oncology education to prepare them to provide better cancer care. Considering the impact of previously employed approaches, researchers believe that adopting diverse and novel education strategies will directly benefit hospital nursing practice. Oncology nursing education will ensure improved proficiency in oncology care, clinical experiences, and learning (74, 75). The currently low oncology education level among Chinese nursing students demands the teaching of basic foundational knowledge and skills for high-quality and effective care for patients across the cancer trajectory (76). Thus, by addressing these insufficiencies in current curricula, educators will positively impact patients and survivors with better-prepared oncology graduates (77).

Regarding the long-term effective education policies on oncology nursing, faculty with oncology backgrounds must develop and revise the curriculum content. In addition, the course design must follow the national guidelines and policies for cancer care (78, 79). Practical oncology nursing also includes research to analyze the most effective methods for incorporating oncology nursing content into national health programs nationwide (80). Finally, the nursing faculty should prioritize the investigation of oncology nursing to provide appropriate evidence on evidence-based practice and policy (81–83).

In China, most of the academic education is to train general nurses. Only through continuing education can the nurse gradually develop along the professional nursing direction and become a professional. All medical institutions cultivate the clinical observation, analysis, and judgment abilities in nurses, broaden their knowledge through pre-job training, and target continuing education and learning on oncology nursing combining their clinical work. These measures will lay the foundation for training specialized nurses (84). Medical institutions can choose suitable personnel training methods according to their actual conditions, such as on-the-job training, advanced education, academic education, and academic exchange. So that nurses can update their knowledge structure, improve their self-quality, exercise their thinking ability, and ultimately become practical talents in oncology nursing (85).

In short, nurses’ presentations in all multidisciplinary teams conducting health services research or clinical trials will expand knowledge about best practices and clinical application of research findings (86). Furthermore, oncology nursing researchers should address the scarcity of China-based studies about oncology education. They should implement oncology education programs and improve the students’ attitudes, confidence, and overall competency in practice in providing oncology nursing care.

### 6.3. Improving the training and qualification certification of oncology nurses and setting up corresponding clinical positions

The concept of oncology nurses needs to be clarified in China. The nurses working in tumor wards of cancer hospitals or general hospitals are often called oncology nurses, but the oncology nurses do not get the authoritative certification. A unified and mature certification system should be established as early as possible after summarizing the domestic experience combined with the current practice of Beijing and other cities and learning from foreign theories and methods (38). Furthermore, medical institutions should improve and provide corresponding clinical positions for oncology nurses who were trained at a high level. Different positions are set up according to oncology nurses’ roles and functions. For example, according to different kinds of diseases in the ward, the specialized nurses for this kind of disease should be primarily trained, such as specialized nurses in breast cancer care and specialized nurses in lung cancer care.

On the other hand, specialized nurses on pain, chemotherapy and radiotherapy, and hospice care can be set up within the hospital or community (87). China can learn from the foreign training mode of the master’s degree in nursing, which is aimed at clinical oncology nurses. The nursing colleges in the university can cooperate with specialized cancer hospitals to cultivate the nursing master to meet the needs of clinical nursing for highly educated specialist nurses in China (88).

### 6.4. Developing community oncology nursing

Palliative care family beds relying on community health service centers are the main form of palliative care home services. This will be emphasized to develop at present and in the future. However, the number of people currently benefiting is very limited in China (89). Because cancer patients need long-term life support and care, community nursing is required to provide patients with long-term care, medical care, psychology, nutrition, physical therapy and other services. However, the current situation is that community nurses require more, the educational quality of community nurses is low, and the residents’ awareness of health care needs to be improved in China.

Moreover, the medical staff’s outpatient fees for family beds, home visits, treatment and accompanying are not covered by the existing medical insurance. Therefore, most patients with advanced cancer and their families have a heavy financial burden. There is indeed a certain difficulty in bearing these costs, which makes it difficult for patients with malignancy to be cared for in the community (90). Therefore, cancer clinics in the community can be set up, and experts in cancer palliative care and traditional Chinese medicine treatment can be hired for regular visits. At the same time, the nursing school can strengthen cooperation with secondary and tertiary hospitals to train relevant nursing talents so that advanced cancer patients, a vulnerable group, can receive adequate care in the community. Furthermore, the health department in China should create a green channel for on-the-job training and continuing education for community medical staff, provide convenience for them to improve their professional skills, and ensure their service quality improves steadily (91).

Moreover, under the guidance of regional health planning, a two-way referral system can be formulated by establishing a counterpart relationship between the community and the hospital. For example, after receiving hospital treatment, patients with advanced cancer are referred to the community to continue receiving supportive treatment and palliative care. They can be transferred to designated hospitals for treatment when their condition changes.

### 6.5. Need for precision medicine training for oncology nurses

In tumor precisive therapy, oncology nurses should seize the opportunity, explore new fields, assess and manage tumor-related symptoms based on precision medicine, and actively participate in all stages of tumor precisive therapy. These include identifying susceptibility genes, and environment-behavioral risk factors, designing effective genetic screening programs, assessing and testing the effectiveness of preventive measures, assessing compliance with health education among at-risk populations, and conducting health surveillance based on genetic susceptibility (28). For example, screening for the BRCA-1 and BRCA-2 genes in breast cancer could have important implications for the early warning of breast cancer. BRCA-1 and BRCA-2 are two tumor suppressor genes that encode proteins repairing damaged DNA to ensure genome integrity. Mutations in these two genes can lead to an increase in cancerous cells. Therefore, oncology nurses can join in the genetic screening of the population with a family history of breast cancer to realize early cancer intervention (92). In addition, regarding gene–environment interactions and symptom-related genetic variation, oncology nursing research can focus on predictive studies of related symptoms, including cancer-related fatigue and pain, and identifying, assessing, and managing individuals at high risk of lymphedema. If precisive oncology nursing can be realized, effective care and rehabilitation programs can be formulated according to the individual characteristics of cancer patients. It can maximize the survival time of patients, avoid excessive intervention, and improve their quality of life (93).

### 6.6. Development of palliative care in oncology nursing in China

**Figure 2:**
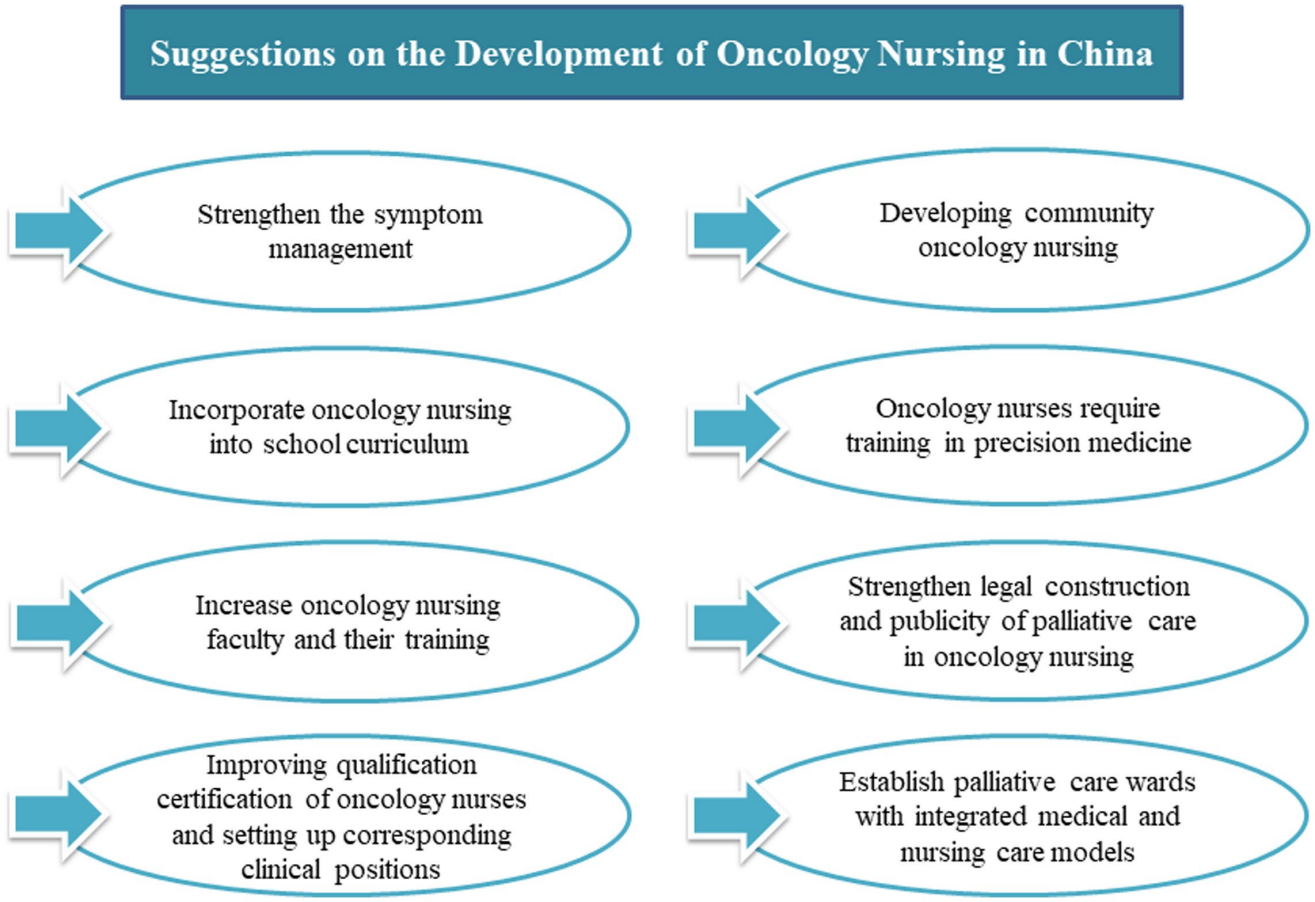
Suggestions on the development of oncology nursing in China.

Firstly, establishing primary palliative care services and making palliative care an independent discipline. Palliative care will run through the whole course of cancer patients, including early treatment, psychological care, pain and symptom management in the middle stage, and hospice care in the later stage. To establish a comprehensive palliative care service system, it is necessary to carry out training for relevant personnel, including care for the uncomfortable symptoms caused by tumor treatment in the early stage and emotional care for patients in the middle and late stages. Secondly, the laws and regulations related to palliative care should be introduced and included in the medical insurance scope. By formulating relevant laws and regulations, the application of palliative care is guaranteed by law. Including palliative care in medical insurance can effectively improve the coverage of palliative care. At the same time, it is necessary to standardize the operation of palliative care, ensure its quality, improve patients’ satisfaction, and promote the healthy development of palliative care (51). Thirdly, doing a good job of publicizing palliative care and strengthening death education is recommended. It encourages patients to change their attitude toward death and accept the diagnosis calmly through various education methods to improve their awareness of palliative care. Fourthly, a palliative care ward with medical and nursing care is recommended. The creation of medical wards that combine nursing homes with palliative care is advancing with the times. This new medical and nursing model can provide life guidance, pain control, medication guidance, psychological counseling and other services for patients in the terminal stage of cancer (52). Figure 2 lists recommendations for establishing oncology nursing in the Chinese healthcare system.

## 7. Conclusion

Nurses are typically the initial point of contact for patients. They are the main component of the healthcare system in China. The most constructive approach to reducing the global burden of cancer is to augment oncology nursing training and learning for comprehensive education. Oncology nursing staff must make urgent and sustained efforts to plan oncology nursing education and training in the Chinese health system. Although China has made significant progress in developing oncology nursing, its healthcare system still faces some problems in oncology nursing that need to be addressed. In the future, it is urgent for the administrative management department and the oncology nurses to make efforts in the following aspects: ①strengthening the symptom management for cancer patients and improving their quality of life; ②strengthening the education of oncology nursing, improving the training and qualification of oncology nurses, setting up corresponding clinical positions; ③ developing community oncology nursing; ④developing precision oncology nursing; and ⑤ Creating palliative care and hospice in most institutions related to oncology nursing. These measures will tremendously promote the development of oncology nursing in China.

## Author contributions

YL, WY, and EJ: conceptualization, writing—original draft; LL, QY, KJ, and TZ: participate in the review and editing; TZ and EJ: writing—original draft, funding acquisition, supervision, validation, and visualization. All authors contributed to the article and approved the submitted version.

## Funding

This work was supported by the Postgraduate Education Reform and Quality Improvement Project of Henan Province (YJS2022KC30 to EJ); the Postgraduate Cultivating Innovation and Quality Improvement Action Plan of Henan University (YJSJG2022XJ059 to EJ); and the Henan Provincial Science and Technology Research Project (222102310251 to TZ).

## Conflict of interest

The authors declare that the research was conducted without any commercial or financial relationships that could be construed as a potential conflict of interest.

## Publisher’s note

All claims expressed in this article are solely those of the authors and do not necessarily represent those of their affiliated organizations, or those of the publisher, the editors and the reviewers. Any product that may be evaluated in this article, or claim that may be made by its manufacturer, is not guaranteed or endorsed by the publisher.
